# Preparation and Electromagnetic Absorption Properties of Fe_73.2_Si_16.2_B_6.6_Nb_3_Cu_1_ Nanocrystalline Powder

**DOI:** 10.3390/ma15072558

**Published:** 2022-03-31

**Authors:** Bingwen Zhou, Mengnan Lv, Jiali Wu, Bin Ya, Linggang Meng, Lanqing Jianglin, Xingguo Zhang

**Affiliations:** 1 School of Materials Science and Engineering, Dalian University of Technology, Dalian 116081, China; lmn@mail.dlut.edu.cn (M.L.); wjl19961028@mail.dlut.edu.cn (J.W.); yabin@dlut.edu.cn (B.Y.); menglg@dlut.edu.cn (L.M.); yingqi@mail.dlut.edu.cn (L.J.); 2 Ningbo Research Institute, Dalian University of Technology, Ningbo 315000, China

**Keywords:** Fe-based nanocrystalline alloy, absorbing performance, soft-magnetic properties, electromagnetic shielding

## Abstract

In order to decrease and control electromagnetic pollution, absorbing materials with better electromagnetic wave absorption properties should be developed. In this paper, a nanocrystalline alloy ribbon with the composition of Fe_73.2_Si_16.2_B_6.6_Nb_3_Cu_1_ was designed and prepared. Nanocrystalline alloy powder was obtained by high-energy ball milling treatment. The effects of ball milling time on the soft magnetic properties, microstructure, morphology, and electromagnetic wave absorption properties of alloy powder were investigated. The results showed that, as time increased, α-(Fe, Si) gradually transformed into the amorphous phase, and the maximum *saturation magnetization* (*M_s_*) reached 135.25 emu/g. The nanocrystalline alloy powder was flakelike, and the minimum average particle size of the powder reached 6.87 μm. The alloy powder obtained by ball milling for 12 h had the best electromagnetic absorption performance, and the minimum reflection loss *RL_min_* at the frequency of 6.52 GHz reached −46.15 dB (matched thickness was 3.5 mm). As time increased, the best matched frequency moved to the high-frequency direction, and the best matched thickness decreased, while the maximum effective absorption bandwidth Δ*f_RL_*_<−10 dB_ was 7.22 GHz (10.78–18 GHz).

## 1. Introduction

With the development of the power electronics industry, the problem of electromagnetic pollution has become more and more serious. In order to solve the increasingly serious electromagnetic pollution problem, it is necessary to develop excellent absorbing materials. Usually, the absorbing material is composed of an absorber and a matrix material, and the absorber is the key to affecting the absorbing performance of the absorbing material. Absorbing materials can also be used in military stealth technology, which is a cutting-edge technology to avoid military radar detection, identification, and tracking strikes. Therefore, the development of absorbing materials with excellent performance is of great practical significance [1,2].

According to their loss mechanism, absorbing materials can be divided into three types: resistive type (silicon carbide, carbon nanotubes, graphene, etc.) [3,4,5,6,7], dielectric loss type (ferroelectric ceramics, MnO_2_, etc.), and magnetic loss type (ferrite [8,9,10,11], carbonyl iron [12,13,14,15,16], magnetic metal alloy powder [17,18,19,20,21,22,23,24,25], etc.). Magnetic alloy powders have been widely studied for their high absorption intensity and wide absorption band. Duan et al. [18] used a high-energy ball milling method to obtain FeCoNi powder with different ball milling times. They found that, with the increase in ball milling time, the wave absorption performance of the powder was enhanced, and the *RL_min_* reached −32.4 dB when the ball milling time was 90 h. Chen et al. [19] and Duan et al. [20] prepared FeSiCr and FeSiAl powders, respectively, and studied the effect of ball milling time on the wave absorption performance. The results showed that the absorbing properties of the alloy were enhanced after ball milling. When *RL_min_* was −41.5 dB and −22.2 dB, Δ*f_RL_*_<−10 dB_ reached 3.6 GHz and 6.6 GHz, respectively. All the above results indicated that the morphology of the alloy can be changed by high-energy ball milling to improve the absorbing properties of the alloy. In addition, the bandwidth properties of the absorbing materials with strong absorption properties also need to be strengthened. Duan et al. prepared FeCoNiCuAl [22] and FeCoNiCrAl high-entropy alloy powder [23] by high-energy ball milling; the *RL_min_* was −47.55 dB, but the effective absorption bandwidth of the two alloys was less than 2.5 GHz. Lan et al. [24] prepared FeCoNiCrCuAl hollow high-entropy alloy powder with an *RL_min_* of −40.2 dB and a Δ*f_RL_*_<−10 dB_ of 4.48 GHz. Zhang et al. [25] obtained a high-entropy FeCoNiCuZn alloy powder with an *RL_min_* of −14.69 dB and a Δ*f_RL_*_<−10 dB_ of 2.5 GHz. The existing materials cannot have both strong absorption and a wide frequency band; therefore, studies should be focused on the development of new absorbing materials.

Fe-based nanocrystalline materials include amorphous and nanocrystalline composite phases. The nano-size grains, well dispersed in the amorphous matrix, can effectively reduce the magnetostrictive coefficient; thus, the soft magnetic properties can be accordingly optimized. Therefore, Fe-based nanocrystalline alloys present excellent soft magnetic properties such as high saturation magnetization *M_S_*, high permeability *μ*, low correction strength *H_C_*, and low iron loss *P*, which make them promising soft magnetic materials [26,27,28,29]. In this regard, Fe-based nanocrystalline alloys are expected to become excellent absorbing materials due to their excellent soft magnetic properties.

According to previous studies, the size and morphology of the powder particles of the absorbing material can greatly influence absorbing properties [18,19,20]. Therefore, in this study, the method of high-energy ball milling was used to control the effect of Fe-based nanocrystalline alloy powder particle size and mechanical ball milling time on the microstructure, morphology, soft magnetic properties, and wave absorption properties of Fe-based nanocrystalline powders. It is well known that obtaining powders via a traditional process (pulverization) can bring many economic benefits, but it should be noted that it is relatively easy to obtain amorphous powders by pulverization, whereas it is not easy to control the proportion of nanocrystalline phase in the powders. In addition, the soft magnetic properties of the powders obtained by pulverization are generally lower than ball-milling samples after spin-casting. Therefore, the excellent performance of Fe-based nanocrystalline absorbing materials can be further developed.

## 2. Experimental Procedures

Fe_73.2_Si_16.2_B_6.6_Nb_3_Cu_1_ alloy ingots were melted by vacuum arc smelting, and the amorphous alloy ribbon (22 μm in thickness) was prepared by a single-roll melt-spinning method. The amorphous alloy ribbon was annealed in vacuum to get Fe_73.2_Si_16.2_B_6.6_Nb_3_Cu_1_ nanocrystalline alloy ribbon. The specific process was as follows: firstly, the amorphous ribbon was heated to 450 °C in a vacuum environment, and the temperature was held for 100 min. Then, the temperature was continuously heated to 530 °C, and the temperature was held for 100 min. Finally, the ribbon-containing nanocrystalline structure was obtained by cooling in the furnace. Then, nanocrystalline alloy powders were prepared by the dry milling method. The nanocrystalline ribbons were put into a stainless-steel vacuum ball milling tank with a ball-to-material ratio of 10:1. The tank was pumped to 8 × 10^−2^ MPa for high-energy mechanical ball milling. The rotating speed was 250 r/min. In this experiment, the ball milling time was set to 6, 8, 10, and 12 h according to the powder yield. Hence, the nanocrystalline alloy powders were obtained after mechanical high-energy ball milling. The saturation magnetization (*M_s_*) and coercivity (*H_C_*) of the powders were measured by a vibrating sample magnetometer (VSM) (Lake Shore 7410) (Lake Shore Company, Westerville, OH, USA). The phase and microstructure of the powders were characterized by X-ray diffraction (XRD-6000, Cu target) (Shimadzu, Japan) and transmission electron microscopy (JEM-2100F) (JEOL, Akishima, Japan). The micromorphology of the powder was observed under a scanning electron microscope (SEM) (JSM-6360LV) (JEOL, Akishima, Japan), and its elements were analyzed by an energy-dispersive spectrometry (EDS). A vector network analyzer (VNA) (8720B) (Keysight, Santa Rosa, CA, USA) was used to measure the absorbing properties of the powder. The mass ratio of the alloy powder to the paraffin wax was 7:3.

## 3. Results and Discussion

**Figure 1:**
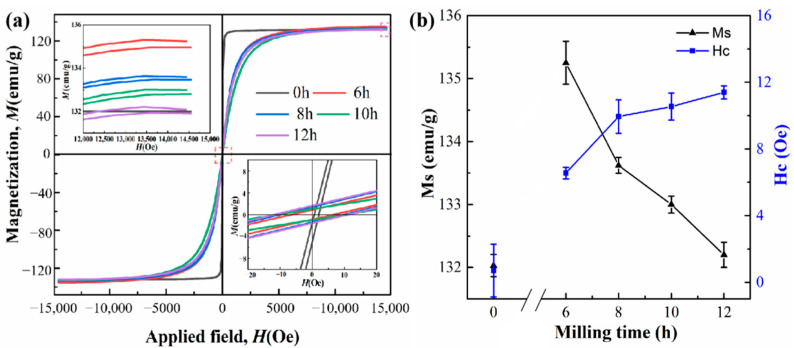
VSM patterns of Fe_73.2_Si_16.2_B_6.6_Nb_3_Cu_1_ nanocrystalline alloy: (**a**) hysteresis loops; (**b**) changes in *M_S_* and *H_C_* as a function of milling time.

Figure 1a shows the VSM results of Fe_73.2_Si_16.2_B_6.6_Nb_3_Cu_1_ nanocrystalline alloy ribbon and powder after ball milling for 6, 8, 10, and 12 h. As can be seen from the figure, the initial *M_S_* of the nanocrystalline alloy ribbon without ball milling treatment was 132.03 emu/g. After 6 h of ball milling treatment, the initial *M_S_* increased to 135.25 emu/g, and then decreased gradually with the increase in ball milling time. After 12 h of ball milling, the initial *M_S_* decreased to 132.20 emu/g. The *H_C_* of the nanocrystalline alloy powder was between 0 and 16 Oe.

**Figure 2:**
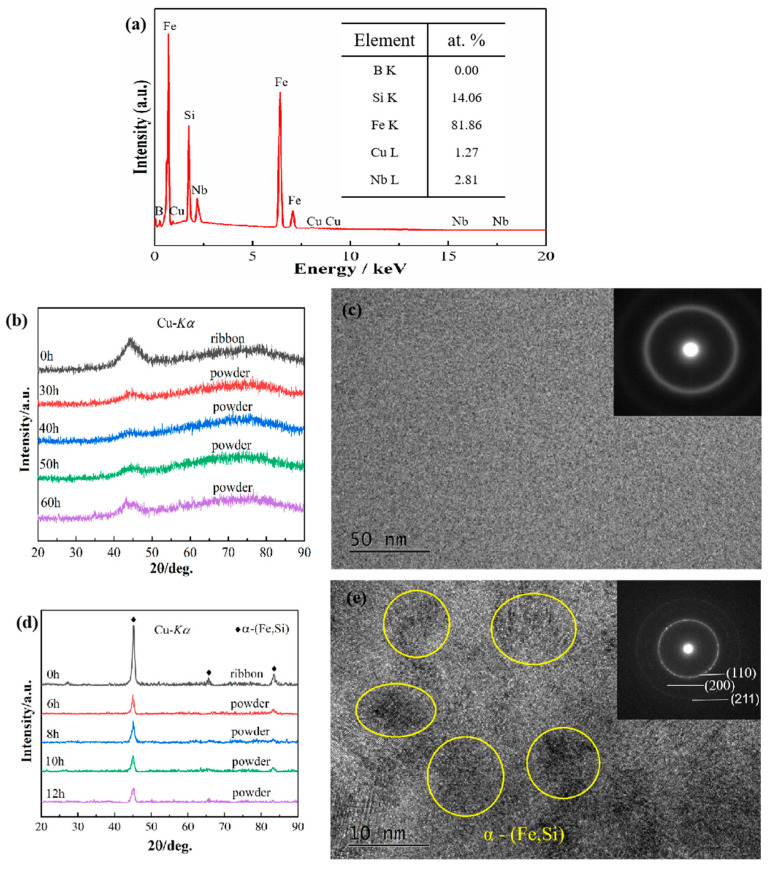
EDS map of Fe_73.2_Si_16.2_B_6.6_Nb_3_Cu_1_ nanocrystalline ribbon, along with XRD patterns and TEM images of Fe_73.2_Si_16.2_B_6.6_Nb_3_Cu_1_ amorphous alloy and nanocrystalline alloy: (**a**) EDS map of the nanocrystalline ribbon; (**b**) XRD patterns of amorphous alloy; (**c**) HRTEM and SAED images of amorphous alloy; (**d**) XRD patterns of nanocrystalline alloy; (**e**) HRTEM and SAED images of nanocrystalline alloy powder after ball milling for 12 h.

In order to study the mechanism of the change in soft magnetic properties, the microstructure of the alloy powder was characterized. Figure 2 shows the EDS map of Fe_73.2_Si_16.2_B_6.6_Nb_3_Cu_1_ nanocrystalline ribbon, as well as the XRD and TEM images of the nonannealed Fe_73.2_Si_16.2_B_6.6_Nb_3_Cu_1_ amorphous alloy and the annealed nanocrystalline alloy. For EDS, the detection range of the element is the element whose atomic number is after the oxygen element. The content of elements with an atomic number lower than that of oxygen cannot be determined. Therefore, the detection of the B element content here is not accurate. According to Figure 2a, the gap between the nominal composition and the actual composition of the comparative analysis material was within an acceptable range; hence, it can be considered that the actual composition of this material was Fe_73.2_Si_16.2_B_6.6_Nb_3_Cu_1_. According to Figure 2b,c, it can be determined that the material was amorphous when not annealed. The XRD image in Figure 2d shows that the diffraction peak of the α-(Fe, Si) phase can be seen in the XRD pattern of the ribbon, indicating that the α-(Fe, Si) phase precipitated on the amorphous matrix after annealing. Furthermore, the diffraction peak of the α-(Fe, Si) phase still existed in the XRD curve of the nanocrystalline alloy powder after 6–12 h ball milling, indicating that the nanocrystalline alloy powder was still a mixed structure of amorphous and nanocrystalline. As time increased, the intensity of the diffraction peak decreased gradually, indicating that the content of α-(Fe, Si) phase decreased. The grain size calculated by the Debye–Scherrer formula is shown in Table 1. As time increased, the diffraction peak gradually became wider, and the size of the nano-grains decreased gradually. Figure 2e shows the TEM images of the nanocrystalline alloy after 12 h ball milling. The crystal structure and amorphous structure can be observed in the high-resolution image. The crystal phase was determined to be the α-(Fe, Si) phase by calibration of the diffraction pattern, confirming that the alloy powder was a mixed structure of amorphous and nanocrystalline phase. The size of the crystal phase was about 10 nm, consistent with the XRD results. The changes in microstructure and structure of the nanocrystalline alloy resulted in a change in the soft magnetic properties of the alloy. According to Table 1, after 6 h of ball milling, large atoms Nb and Cu were gradually dissolved in the α-(Fe, Si) phase, and the crystal phase was gradually transformed into the amorphous phase, resulting in a larger lattice constant and a larger shift in the diffraction peak of the crystal phase to a lower angle. According to the Bethe–Slater curve, a larger atomic spacing causes an increase in Ms. Therefore, the M_S_ of the powder increased after 6 h of ball milling and decreased gradually with the increase in ball-milling time from 6 h to 12 h. This is because the content of the α-(Fe, Si) soft magnetic phase in the alloy gradually decreased with the increase in ball milling time. Moreover, the amorphous phase had a lower *M_S_*; hence, the *M_S_* of the alloy decreased. As time increased, *H_C_* of nanocrystalline alloy powder showed an increasing trend, which was due to the increase in the internal stress of the alloy caused by the ball milling treatment.

**Figure 3:**
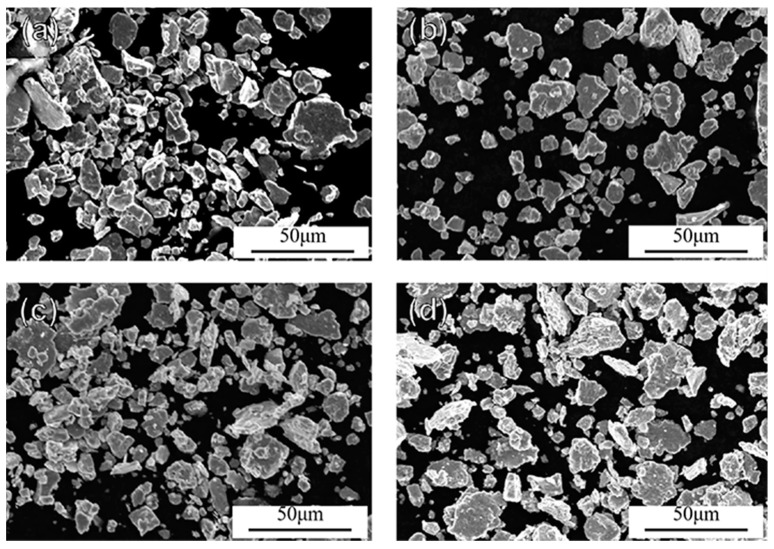
SEM images of Fe_73.2_Si_16.2_B_6.6_Nb_3_Cu_1_ nanocrystalline alloy powder after milling for (**a**) 6 h, (**b**) 8 h, (**c**) 10 h, and (**d**) 12 h.

**Figure 4:**
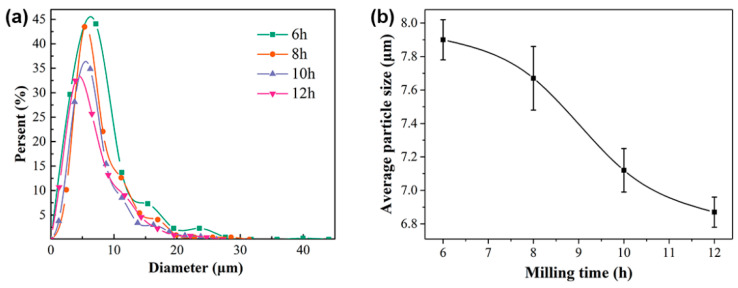
Particle size of Fe_73.2_Si_16.2_B_6.6_Nb_3_Cu_1_ nanocrystalline alloy powder: (**a**) particle size distribution; (**b**) change in average particle size as a function of milling time.

Figure 3a–d show the SEM images of nanocrystalline alloy powders after 6 h, 8 h, 10 h, and 12 h ball milling, respectively. It can be observed from the figure that the nanocrystalline alloy powders were flat sheets. According to the Snoek limit principle [30], the absorbent with flake morphology can more easily obtain better absorbing performance. The particle size of the nanocrystalline powder was statistically analyzed, and the curve of particle size distribution with ball milling time is shown in Figure 4a. It can be seen that the particle size distribution of the nanocrystalline alloy powder ranged from 1 to 45 μm. As time increased, the particle size of the powder gradually decreased to a smaller size. Furthermore, the average particle size of the nanocrystalline alloy powder decreased with the increase in ball milling time, from 7.90 μm after 6 h to 6.87 μm after 12 h, as shown in Figure 4b.

**Figure 5:**
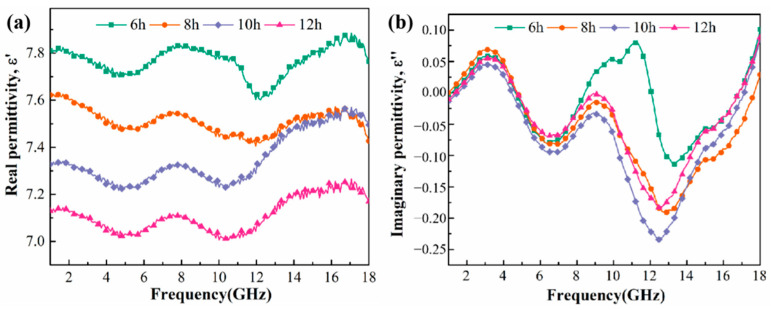
Frequency dependences of *ε*′ (**a**) and *ε*″ (**b**) of Fe_73.2_Si_16.2_B_6.6_Nb_3_Cu_1_ nanocrystalline alloy powder.

The electromagnetic wave absorption performance is evaluated mainly through the alternating field of the material of the complex dielectric constant (*ε_r_ = ε*′ − *jε*″) and complex permeability (*μ_r_ = μ*′ − *jμ*″). The real part (*ε*′ and *μ*′) and the imaginary part (*ε*″ and *μ*″) reflect the storage capacity and extinguish extent of the electromagnetic energy for certain materials, where a larger value indicates stronger storage or extinguish performance. The complex dielectric constant curve of Fe_73.2_Si_16.2_B_6.6_Nb_3_Cu_1_ nanocrystalline alloy powder is shown in Figure 5. The *ε*′ value of the nanocrystalline alloy was 7.00–7.88, while *ε*″ was −0.24–0.08. It can also be observed from the figure that *ε*′ and *ε*″ of the nanocrystalline alloy powder fluctuated as frequency increased. With the increase in milling time, *ε*′ and *ε*″ showed a downward trend, because both the content and the size of α-(Fe, Si) were gradually reduced; thus, the crystal phase changed to an amorphous phase, and the interface between crystalline and amorphous phases was reduced. Accordingly, the interfacial polarization was abated, and the polarization loss was reduced.

**Figure 6:**
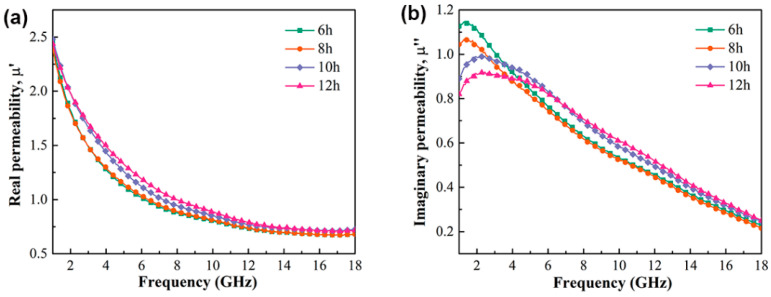
Frequency dependences of *μ*′ (**a**) and *μ*″ (**b**) of Fe_73.2_Si_16.2_B_6.6_Nb_3_Cu_1_ nanocrystalline alloy powder.

Figure 6 shows the complex permeability curve of Fe_73.2_Si_16.2_B_6.6_Nb_3_Cu_1_ nanocrystalline alloy powder. It can be observed from the figure that *μ*′ of the nanocrystalline alloy powder ranged from 0.67 to 2.45, while *μ*″ ranged from 0.21 to 1.15. The nanocrystalline alloy powders had a natural resonance peak near 1.5–2 GHz, and the resonance frequency was less than 10 GHz; therefore, this can be considered as a natural resonance peak. Nanocrystalline alloy powder also showed a strong frequency dependence. With the increase in frequency, the eddy current loss and skin effect increased, resulting in a low powder absorbing performance. With the increase in milling time, the powder particle size decreased, inhibiting the eddy current loss of powder particles. Therefore, with the increase in ball milling time, the powder particle size decreased, while *μ*″ increased at high frequency, indicating that ball-milling treatment could effectively improve the high-frequency wave absorption performance of the nanocrystalline alloy powder. Moreover, with the increase in milling time, the particle size of the alloy decreased gradually, and the magnetic exchange coupling between the nanocrystalline alloy powders increased, leading to higher *μ*′. However, the maximum value of *μ*″ decreased gradually because the complex permeability of the alloy is positively correlated to the square value of *M_S_*, as shown in Equation (1).

(1) $$\mu_{i}\approx \frac{\mu_{0}M_{s}^{2}}{\left(K_{1}+\frac{3}{2}\lambda_{s}\sigma \right)\beta^{1/3}\frac{\delta }{d}},$$

where *μ_i_* is the initial permeability, *μ*_0_ is the free-space permeability, *K*_1_ is the magnetocrystalline anisotropy coefficient, *λ_S_* is the magnetostriction coefficient, *σ* is the internal stress density, *β* is the volume fraction of impurities, *δ* is the domain wall thickness, and *d* is the particle size of impurities. Thus, *μ*″ obeys the same law as *M_S_*.

**Figure 7:**
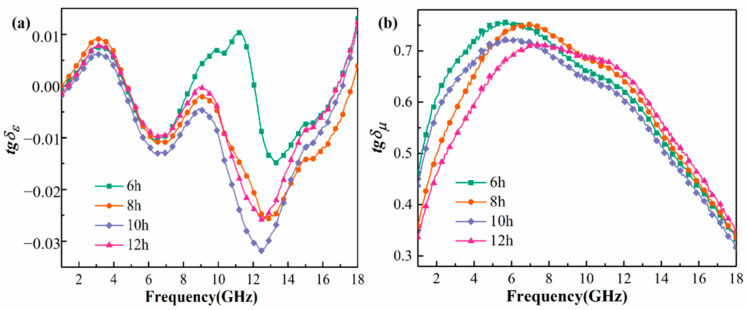
Frequency dependences of *tgδ_ε_* (**a**) and *tgδ_μ_* (**b**) of Fe_73.2_Si_16.2_B_6.6_Nb_3_Cu_1_ nanocrystalline alloy powder.

Figure 7 shows the variation curve of the dielectric loss tangent angle *tgδ_ε_* and magnetic loss tangent angle *tgδ_μ_* with frequency of Fe_73.2_Si_16.2_B_6.6_Nb_3_Cu_1_ nanocrystalline alloy powder milling (6–12 h). It can be seen that the *tgδε* of nanocrystalline alloy powder ranged from −0.03 to 0.01, while *tgδ_μ_* ranged from 0.32 to 0.76. *tgδ_μ_* was much larger than *tgδε*, indicating a strong magnetic loss property; hence, this is a magnetic loss absorbing material. *tgδ_μ_* first increased and then decreased with the increase in frequency. The peak value of *tgδ_μ_* was between 5.5 GHz and 7.8 GHz. With the increase in ball milling time, *tgδ_μ_* in the high-frequency band increased gradually. The peak of *tgδ_μ_* gradually moved in the high-frequency direction, indicating that the high-frequency absorption performance was improved.

**Figure 8:**
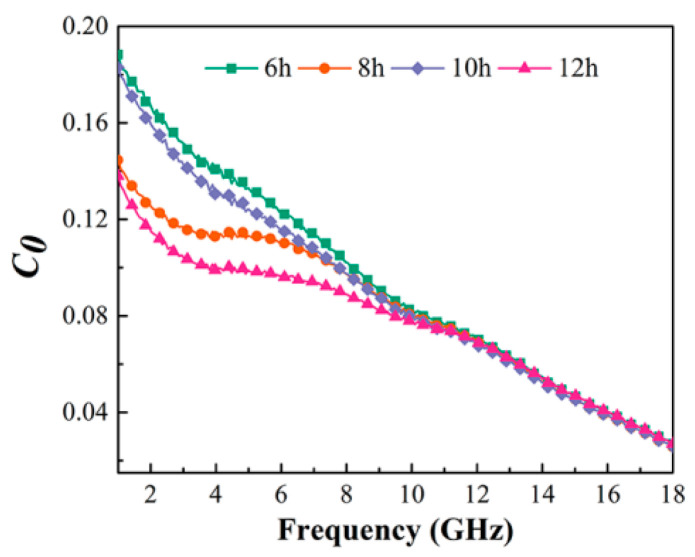
Frequency dependence of *C*_0_ of Fe_73.2_Si_16.2_B_6.6_Nb_3_Cu_1_ nanocrystalline powder.

The magnetic loss of materials is mainly caused by hysteresis loss, domain wall resonance, natural resonance, and eddy current loss. The hysteresis loss in the weak electromagnetic field can be ignored, while the domain wall resonance only appears at low frequency (<2 GHz); thus, the magnetic loss in the range of gigabits mainly includes two forms: natural resonance and eddy current loss. Eddy current losses can be expressed as shown in Equation (2), where *f*, *σ*, and *d* represent the frequency, conductivity, and absorber thickness, respectively. If the magnetic loss of the material is only caused by eddy current loss, then the value of *C*_0_ should remain constant over all frequency bands. The *C*_0_ value of Fe_73.2_Si_16.2_B_6.6_Nb_3_Cu_1_ nanocrystalline alloy powder is shown in Figure 8. It can be seen that the C_0_ value of the nanocrystalline alloy powder decreased with the increase in frequency, indicating that the magnetic loss of the nanocrystalline alloy included eddy current loss and natural resonance. Among them, the formant of natural resonance appeared near 1.5–2 GHz.

(2) $$C_{0}=\mu^{\prime \prime }{\left(\mu^{\prime }\right)}^{-2}f^{-1}=2\pi \mu_{0}\sigma d^{2}/3.$$

**Figure 9:**
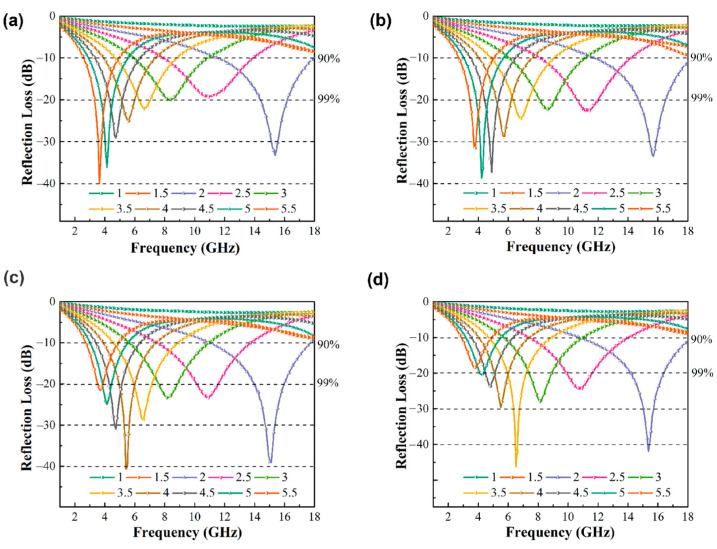
Reflection loss of Fe_73.2_Si_16.2_B_6.6_Nb_3_Cu_1_ nanocrystalline alloy powder after milling for (**a**) 6 h, (**b**) 8 h, (**c**) 10 h, and (**d**)12 h in the frequency range of 2–18 GHz.

**Figure 10:**
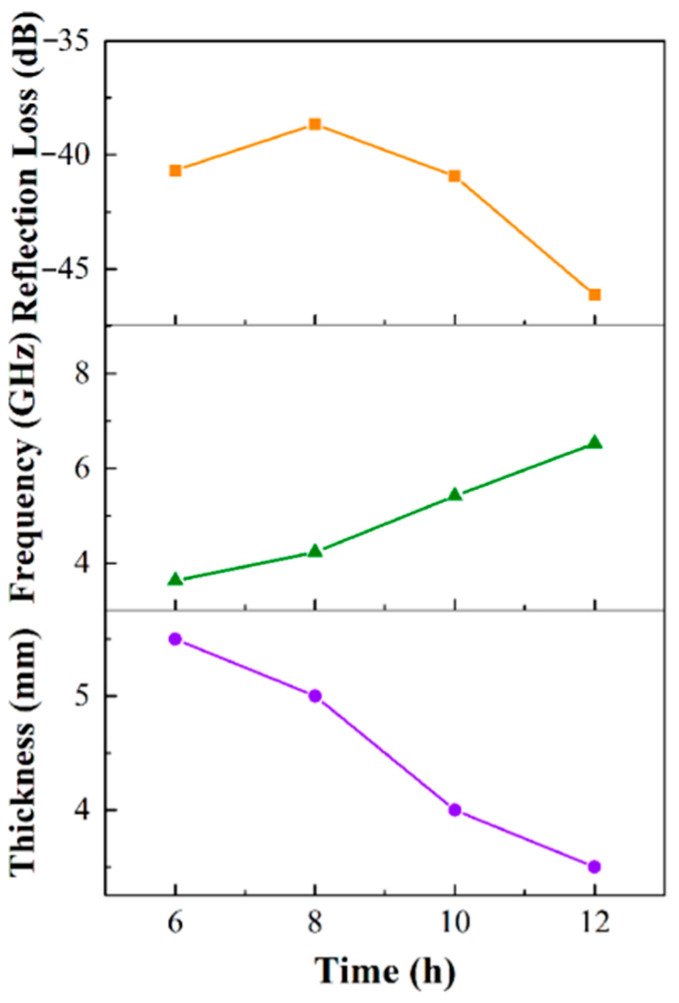
Curves of the minimum reflection loss *Rl_min_*, the best matching frequency *f_Rlmin_*, and the best matching thickness *d_Rlmin_* of Fe_73.2_Si_16.2_B_6.6_Nb_3_Cu_1_ nanocrystalline alloy powder as a function of ball milling time.

The reflection loss *RL* of the alloy can be calculated according to the transmission line principle, as shown in Equations (3) and (4), where *Z*_0_ is the wave impedance in free space, *Z_in_* is the dielectric wave impedance, *f* is the frequency of the incident electromagnetic wave, *c* is the speed of light (3 × 10^8^ m/s), and *d* is the thickness of the absorbent (mm). Figure 9 shows the wave absorption curve of Fe_73.2_Si_16.2_B_6.6_Nb_3_Cu_1_ nanocrystalline alloy powder. It can be seen that the reflection loss of the fixed thickness of the nanocrystalline alloy powder decreased first and then increased with the increase in frequency. It featured an absorption peak, and the minimum reflection loss *Rl_min_* could be obtained for the alloy powder at a specific thickness. Absorbers that are too thin or too thick will absorb electromagnetic waves differently due to the effect of impedance matching. The minimum reflection loss of nanocrystalline alloy powder after 6–12 h ball milling was about −40 dB. As shown in Figure 10, the minimum reflection loss *Rl_min_* of Fe_73.2_Si_16.2_B_6.6_Nb_3_Cu_1_ nanocrystalline alloy powder and the corresponding frequency and thickness of *Rl_min_* (i.e., the best matched frequency *f_Rlmin_* and the best matched thickness *d_Rlmin_*) changed with ball milling time. *Rl_min_* increased first and then decreased with the increase in ball milling time, reaching the minimum value of −46.15 dB after 12 h of ball milling. The *f_Rlmin_* of the alloy powder moved in the high-frequency direction with the increase in milling time, from 3.64 GHz for 6 h to 6.52 GHz for 12 h. Because the particle size of the alloy powder decreased, the skin effect was weakened, and the negative effect of eddy current loss on the high-frequency wave absorption performance was weakened. The *d_Rlmin_* of the alloy powder decreased with the increase in milling time, which was conducive to the lightweight design of the absorber. The minimum matching thickness reached 3.5 mm after 12 h of ball milling.

(3) $$RL\left(\mathrm{dB}\right)=20\log\left|\frac{Z_{in}-Z_{0}}{Z_{in}+Z_{0}}\right|.$$

(4) $$Z_{in}=Z_{0}\sqrt{\frac{\mu }{\varepsilon }}tan\left[j\left(\frac{2\pi fd}{c}\right)\sqrt{\mu \varepsilon }\right].$$

**Figure 11:**
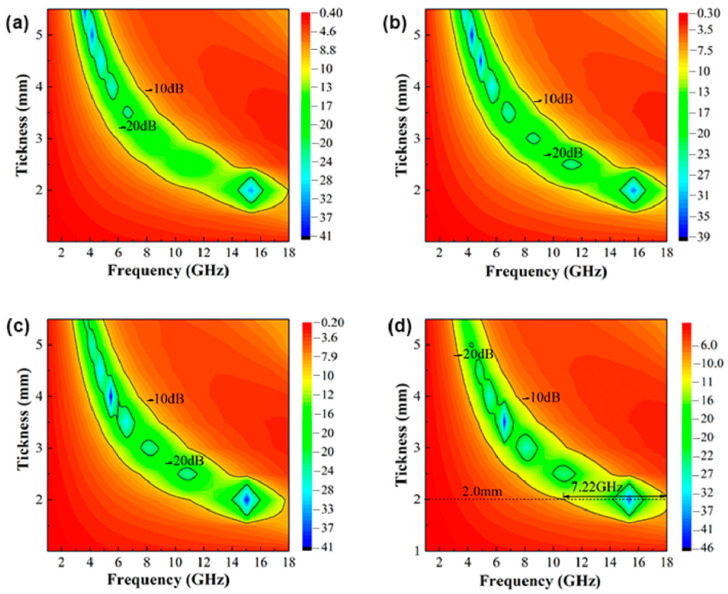
Contour diagram of reflection loss of Fe_73.2_Si_16.2_B_6.6_Nb_3_Cu_1_ nanocrystalline alloy powder as a function of thickness and frequency after milling for (**a**) 6 h, (**b**) 8 h, (**c**) 10 h, and (**d**) 12 h.

Figure 11a–d show the contour plot of reflection loss of Fe_73.2_Si_16.2_B_6.6_Nb_3_Cu_1_ nanocrystalline alloy powder after ball milling for 6–12 h as a function of thickness and frequency. The contour lines in the figure represent 90% effective absorption below −10 dB and 99% absorption below −20 dB, respectively. It can be seen that the nanocrystalline alloy powder of ball milling for 12 h had the best bandwidth performance, and the effective absorption bandwidth was higher below −10 dB when the thickness of the absorbent was 2 mm, while Δ*f_RL_*_<−10 dB_ was up to 7.22 GHz (10.78–18 GHz), covering nearly half of the X-band and all of the Ku band.

## 4. Conclusions

(1) After ball milling, the nanocrystalline alloy remained an amorphous–nanocrystalline mixed structure. With the increase in ball milling time, α-(Fe, Si) gradually transformed into the amorphous phase, and the maximum *M_s_* reached 135.25 emu/g.

(2) The nanocrystalline alloy powder after ball milling was flakelike. The minimum average particle size of the powder reached 6.87 μm. The decrease in particle size weakened the skin effect caused by eddy current loss and enhanced the absorption performance of high-frequency electromagnetic waves.

(3) Nanocrystalline alloy powders had excellent electromagnetic absorption properties. The real part *μ*′ of the complex permeability ranged from 0.60 to 1.97, and the imaginary part *μ*″ and *tgδ_μ_* reached the maxima of 1.15 and 0.76, respectively. The alloy powder obtained from ball milling for 12 h had the best electromagnetic absorption performance, and the minimum reflection loss *RL_min_* at the frequency of 6.52 GHz reached −46.15 dB (matched thickness = 3.5 mm).

(4) With the increase in ball milling time, the best matched frequency moved to a higher frequency, and the best matched thickness decreased. When the thickness of the absorbent was 2 mm, the maximum effective absorption bandwidth Δ *f_RL_*_<−10 dB_ was 7.22 GHz (10.78–18 GHz).

## Figures and Tables

**Table 1:** Grain size of nanocrystalline alloy with different milling time.

Milling Time (h)	Peak Center (°)	Width of Half Height (°)	Grain Size (nm)
0	45.12	0.58	14.72
6	45.00	0.62	13.76
8	45.12	0.63	13.46
10	45.08	0.64	13.37
12	45.31	0.67	12.74

## Data Availability

The raw/processed data required to reproduce these findings cannot be shared at this time as the data also form part of an ongoing study.
